# Modulation of Immune Responses by Particle Size and Shape

**DOI:** 10.3389/fimmu.2020.607945

**Published:** 2021-02-12

**Authors:** Maksim V. Baranov, Manoj Kumar, Stefano Sacanna, Shashi Thutupalli, Geert van den Bogaart

**Affiliations:** ^1^ Department of Molecular Immunology and Microbiology, Groningen Biomolecular Sciences and Biotechnology Institute, University of Groningen, Groningen, Netherlands; ^2^ Simons Center for the Study of Living Machines, National Centre for Biological Sciences, Tata Institute for Fundamental Research, Bangalore, India; ^3^ Molecular Design Institute, Department of Chemistry, New York University, New York, NY, United States; ^4^ International Centre for Theoretical Sciences, Tata Institute for Fundamental Research, Bangalore, India

**Keywords:** phagocytosis, F-actin (filamentous actin), pathogens, vaccination, endocytosis

## Abstract

The immune system has to cope with a wide range of irregularly shaped pathogens that can actively move (e.g., by flagella) and also dynamically remodel their shape (e.g., transition from yeast-shaped to hyphal fungi). The goal of this review is to draw general conclusions of how the size and geometry of a pathogen affect its uptake and processing by phagocytes of the immune system. We compared both theoretical and experimental studies with different cells, model particles, and pathogenic microbes (particularly fungi) showing that particle size, shape, rigidity, and surface roughness are important parameters for cellular uptake and subsequent immune responses, particularly inflammasome activation and T cell activation. Understanding how the physical properties of particles affect immune responses can aid the design of better vaccines.

## Introduction

**Figure 1:**
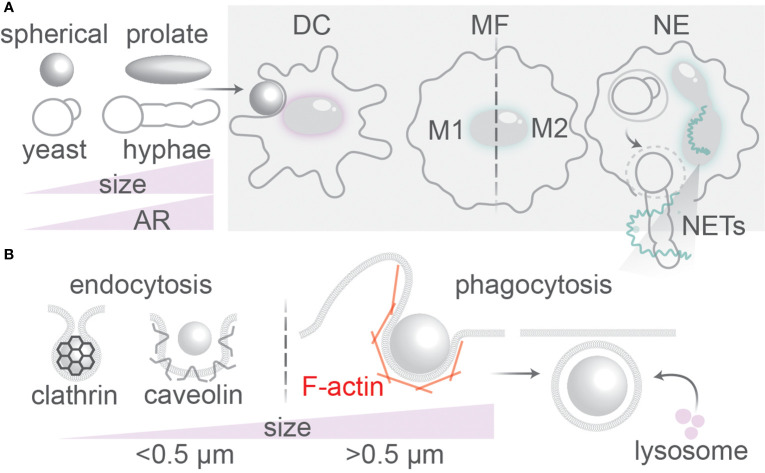
Uptake of antigenic particles by immune phagocytes. **(A)** Dendritic cells (DC), macrophages (MF), and neutrophils (NE) of the immune system encounter particles with different physical properties (size, shape, rigidity, surface roughness). NE can release extracellular traps (NETs) for capturing large pathogens such as hyphal fungi. Macrophages can undergo reprogramming into pro-inflammatory (M1) or anti-inflammatory (M2) phenotypes. **(B)** Particles >0.5 µm are ingested by phagocytosis; smaller particles by endocytosis which can be clathrin or caveolin-mediated. The F-actin cytoskeleton is important for membrane wrapping at the phagocytic cup. Fusion with lysosomes containing lytic enzymes is the final step in endo/phagosomal maturation.

The cells of immune system can morphologically change their plasma membrane for uptake and clearance of foreign particles that enter the body, such as bacteria and fungi upon an infection, and particles from endogenous sources, such as apoptotic cell bodies (1). The cellular uptake of extracellular particles is essential for many cellular functions, and, among other things, plays important roles in the immune system and for tissue remodeling. Pathogens or particles entering the body are ingested by immune phagocytes by engaging receptors on their surface. This process is required for the clearance of infectious microbes (e.g., bacteria, fungi), senescent (cancer) cells and inorganic particles from the body by immune phagocytes (1). Endocytosis is an umbrella term for different types of uptake of smaller particles, while larger particles are taken up by a mechanistically different mechanism called phagocytosis. In this review we will define endocytosis as the uptake of particles < 0.5 μm in size and phagocytosis as the cellular uptake of particles larger than 0.5 μm. Endocytic vesicles can have vesicular, flask-like, or tubular morphologies (2). The best understood form of endocytosis is mediated by clathrin, a coat protein on the cytoplasmic side of the membrane, but other coat proteins (e.g., caveolin) also exist and endocytosis seems not always dependent on coat proteins (2). Due to size constrains of the clathrin lattice, clathrin has a preference for orchestrating uptake of small particles (<0.1 μm in diameter). Membrane invaginations, originating from membrane ruffles and from actin-rich membrane extensions called lamellipodia, form phagocytic cups, which upon sealing lead to formation of large μm-sized membrane-bound vacuoles called phagosomes (receptor-mediated process) or macropinosomes (receptor-independent) (2, 3). Phagocytosis is generally mediated by the actin cytoskeleton that provides a substantial force for overcoming physical constrains necessary for the membrane wrapping of larger particles (**Figure 1**). Phagocytosis differs from the uptake of smaller particles by endocytosis, as it does not rely on coat proteins, such as clathrin and caveolin, and endocytosis has less involvement of the actin cytoskeleton (1, 2). For actin polymerization, phagocytosis requires activity of Rho-GTPases and phosphoinositide 3-kinase (PI3K)-family kinases (2), and this is especially necessary for phagocytosis of larger particles (> 5 µm) (4, 5). In this review, the focus is on μm-range model particles and fungal pathogens, as the effects of morphology on cellular uptake are best understood for these phagocytic cargoes.

The main immune phagocytes are macrophages, neutrophils and dendritic cells (DCs) (1). Neutrophils are the most abundant phagocytes in the body, capable of eliciting oxidative and non-oxidative microbicidal responses, such as by secretion of antibacterial proteins and trapping of pathogens in neutrophil extracellular traps (NETs) of released DNA (**Figure 1**) (6). Macrophages are found in all tissues and are able to clear infections by phagocytosis and trigger inflammatory responses. They are more equipped to phagocytose smaller morphological states of fungi; larger hyphae or spores sometimes cannot be taken up by macrophages (7–9). Macrophages can polarize into pro-inflammatory (M1) phenotypes if activated by pro-inflammatory stimuli such as interferon (IFN)-γ, granulocyte-macrophage colony-stimulating factor (GM-CSF), or microbial stimuli such as lipopolysaccharide (LPS) (10). Alternatively, macrophages can polarize into anti-inflammatory homeostatic (M2) phenotypes induced by anti-inflammatory cytokines interleukin (IL)-4 or IL-13 (10). The shift from M2 to M1-like phenotypes is associated with more microbicidal activity of macrophages (10). Similar to neutrophils and macrophages, dendritic cells (DCs) also sample the blood and peripheral tissues of the body for pathogens and apoptotic/necrotic cells (11). However, in contrast to neutrophils and macrophages, DCs can efficiently migrate to the lymph nodes after phagocytosis of pathogens. In the lymph nodes, the DCs can present antigenic peptides derived from the ingested pathogenic proteins on their cell surface in major histocompatibility complex (MHC) class II to naive CD4-positive “helper” T cells (Th) and, in a process called cross-presentation, in MHC class I to naive CD8-positive “killer” T cells (11, 12). Antigen (cross-)presentation by DCs is essential to activate naive T cells and elicit an adaptive immune response against invading pathogens and cancer.

Because clearance of different pathogens (fungi, bacteria, viruses, helminths) and malignant cells requires different immune responses, the immune system has evolved many receptors that recognize different pathogens, called pathogen recognition receptors (PRR). These PRRs recognize so-called microbial-associated molecular patterns (MAMPs), which are structures present on microbes but not host cells, and danger-associated molecular patterns (DAMPs), which are present on damaged but not (or only in low amounts) on healthy host cells (13). Many PRRs, but not all, can also trigger endocytosis and/or phagocytosis. For example, the fungal cell wall β-glucans and chitins trigger both antigen recognition and phagocytosis by binding to the C-type lectins dectin-1 and the mannose receptor (CD206) on the surface of DCs and macrophages. A major class of PRRs are Toll-like receptors (TLR), which bind to many different MAMPs, such as TLR4 which binds to LPS. The engagement of different receptors, such as dectin-1 and TLRs, enables immune phagocytes to differentiate between different morphological states of fungi (14, 15). For example, compared to the hyphae, the swollen conidia form of the pathogenic fungus *Aspergillus fumigatus* has increased levels of surface-exposed β-glucans and triggers stronger inflammatory responses (16).

Likely because different pathogens have different sizes and engage different phagocytic PRRs, it can trigger different types of phagocytosis. For example, large hyphal forms of the mold *Aspergillus fumigatus* are ingested by Fc-receptor mediated phagocytosis, whereas smaller spores of the same mold are taken up *via* the mannose receptor (10). Phagocytosis mediated by the Fc-receptor triggers a so-called zipper model of uptake, characterized by a close-fitting zipper-like advance of membrane and the branched actin cytoskeleton over the particle surface; the efficiency of this process depends on the particle rigidity, size, the density of antibodies on the particle surface (17–19) and the distance between the phagocyte’s membrane and the particle surface (limited by the size of the Fc-receptor-ligand complex) (20). Less stiff apoptotic cells are taken up through the zipper model as well (3), but with less efficiency than stiffer targets such as bacteria and yeasts (17). In contrast, phagocytosis by complement receptor 3 (CR3) occurs *via* flappy membrane ruffles orchestrated by linear actin, smaller membrane protrusions and phagosomal “sinking” into the cytoplasm (reviewed in (3)). Recent data shows that Fc- and complement-mediated uptake can be intertwined and both can rely on the anchoring of actin *via* integrin α_M_β_2_ at the leading edge of pseudopods at the phagocytic cup (17, 21).

Particles that are too large for ingestion by immune phagocytes result in stalling of the phagocytic process and this is called frustrated phagocytosis (22, 23). Such frustrated phagocytosis can result in the so-called fiber paradigm that sets out the basis for the harmful effects of long (>15 µm), thin, non-biodegradable fibers such as asbestos in the lung (22). Here, the alveolar macrophages can only partially take up the fiber, resulting in the release of lytic enzymes and reactive oxygen species (ROS) in the extracellular environment (22). This release of these contents can lead to inflammation and tissue damage, and eventually can trigger chronic inflammation, fibrosis, and oncogenesis (22, 24).

Phagocytosis and endocytosis thus depend on the type of PRR and phagocytic receptor engaged, but also on the geometry and mechanical properties of the particle (23, 25–34). How the morphology of particles affects their uptake is well-understood from experimental (23, 25–33) and theoretical studies (34–41). The goal of this review is to draw general conclusions on how geometry and mechanical properties affect endocytosis and phagocytosis and the subsequent endo/phagosomal maturation and immune responses, and on how this knowledge can be used in particle-based vaccine design. We therefore compared both theoretical and experimental studies with differently sized and shaped model particles and pathogens. However, the conclusions from these comparisons have to be interpreted with care, because the modes of uptake can be very different among cells types and the direct comparison between endocytic and phagocytic cargoes and techniques might not always be warranted.

## Particle Size and Shape: Theoretical Studies and Modeling

**Figure 2:**
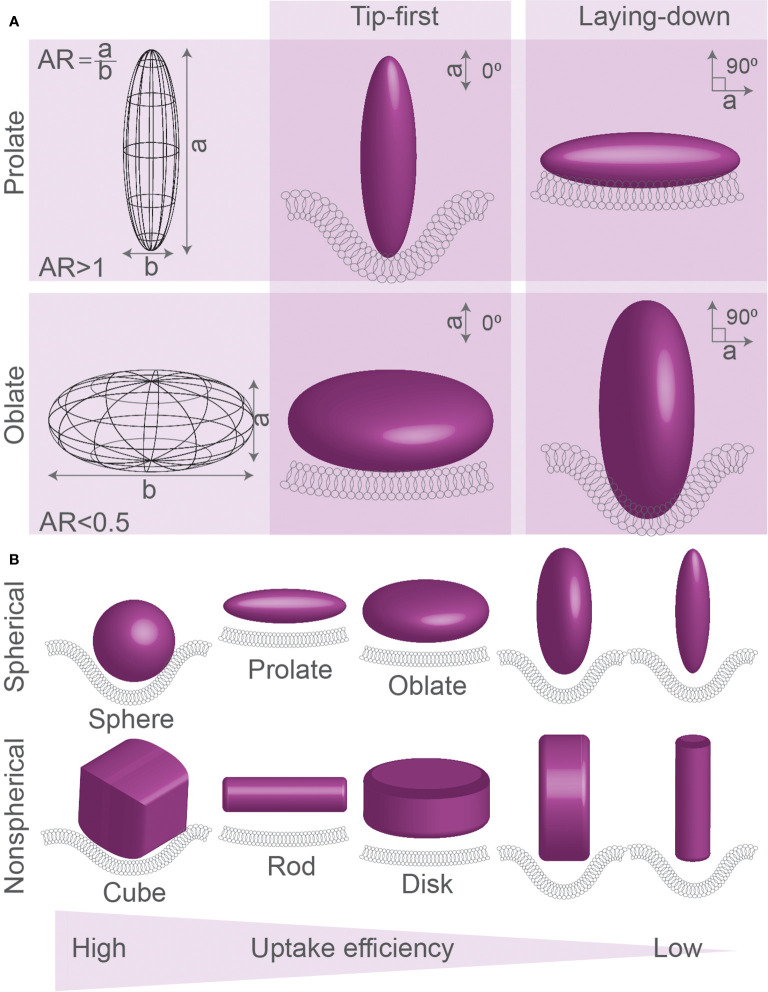
Spherical, oblate, and prolate particles. **(A)** Spheroid particles can be described by their aspect ratio (AR), which is the ratio between the ellipsoid axis (axis a) over the spherical axis (axis b) of the particle: oblate disk-like particles (AR<1), spheres (AR=1), and prolate particles (AR>1). Particles can be ingested by different modes of uptake: tip-first (membrane first faces highest curvature of prolate particles or lowest curvature faces of oblate particles) or laying-down (membrane first faces lowest curvature of prolate particles or highest curvature faces of oblate particles). **(B)** Simulations of uptake of spherical, prolate, oblate, and corresponding non-spherical particles (cubic, rod-like, and disc-like) generally predict that spherical particles are ingested better than prolate and oblate particles. Prolate particles are generally predicted to be ingested most efficiently in laying-down mode, whereas oblate particles are ingested best in tip-first mode. Details; see text.

**Figure 3:**
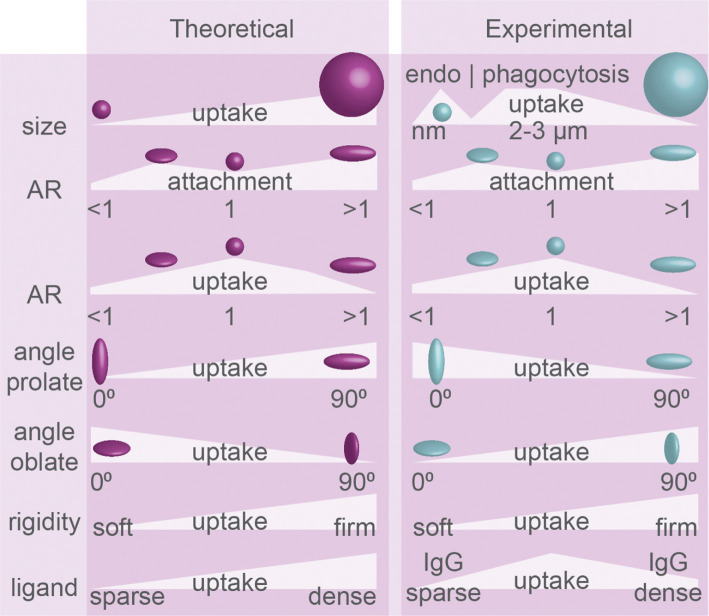
Uptake efficiency determined by physical properties of particles: theoretical predictions and experimental evidence. Particle geometry in phagocytosis. Size, aspect ratio (AR), angle of initial contact between principle axis and membrane (see **Figure 2**), particle rigidity, and ligand density dictate the efficiency of endo/phagocytosis. Summary of theoretical predictions and experimental results. The experimental evidence for how particle rigidity affects uptake is controversial.

Theoretical approaches have been extensively used to predict modes of uptake for different particles with different sizes and shapes (**Supplementary Table 1**) (35–37, 41). Spheroid particles can be described by their aspect ratio (AR), which is the ratio between its ellipsoid axis (axis a, see **Figure 2**) over its spherical axis (axis b): oblate disk-like particles (AR<1), spheres (AR=1), and prolate particles (AR>1). Prolate particles with high AR (AR>2) are also called ellipsoid or rod-shaped particles. Monte-Carlo (35) and coarse-grained (39) simulations compared three modes of uptake for oblate particles and ellipsoids: tip-first (membrane first faces highest curvature of prolate particles or lowest curvature faces of oblate particles), laying-down (membrane first faces lowest curvature of prolate particles or highest curvature faces of oblate particles), and tilted (principle axes are non-perpendicular relative to the membrane) (**Figure 2**). From these simulations, it was predicted that, at least for endocytosis of small particles with sizes between 25 and 100 nm, oblate particles with a low AR (AR<0.5) are likely to be ingested by tip-first uptake mode, e.g., facing the membrane with the lowest curvature side (35, 39) (**Figure 3**). Likewise, prolate particles are more likely to be ingested by laying-down mode, thus also facing the membrane with the lowest curvature side (39). Other computational studies also predicted that prolate particles are more likely to be ingested *via* the laying-down mode (35, 37) and that tip-first uptake for prolate particles will be slower than tip-first uptake for oblate particles (34). However, the wrapping can be accompanied by a reorientation of the particle. For instance, in several theoretical studies, it was predicted that while prolate particles become attached to the membrane in a laying-down mode, at higher adhesive strengths this state becomes unstable and the particle reorients toward a deeply wrapped tip-first uptake mechanism (42, 43).

The energy of uptake depends on the bending properties of the membrane and the strength of receptor interactions, and overall endocytosis is more efficient (lower free energy requirement) with a stronger adhesiveness and a larger contact area (44). Simulations of clathrin-mediated endocytosis of ~100 nm particles predicted that the efficiency of uptake is determined by the membrane bending rigidity, which is proportional to the stiffness of the plasma surface because this determines the formation of receptor-ligand bonds (37). Computational modelling also predicted that a membrane can completely wrap particles with 0.68<AR<2.3 (35). Compared to spherical prolate particles, more stretched ellipsoid particles of the same volume can be less efficiently ingested because the reduced contact area facilitates less ligand-receptor interactions (35). Because of this, spheres are predicted to be engulfed more efficiently than prolate and oblate ellipsoids (**Figure 3**) (38), although particles with a bit of oblate shape, but close to spherical, might have an optimal contact area and therefore be engulfed faster than precisely round spheres (34). Thus, computational modelling predicts hindered uptake of particles with extreme shapes characterized by either high or low ARs, while more spherical particles are more permissive for uptake (**Figure 3**) (35, 38, 39).

Simulations show that for particles of different shapes and volumes, the membrane has to overcome different free energy thresholds for particle wrapping (38, 45). Because of an increased energy cost for wrapping of the highly curved tips, ellipsoid particles are predicted to be ingested less efficiently than similarly sized spherical particles (45). Due to the lower local curvature of larger particles, smaller volume oblate particles are predicted to be taken up less efficiently than smaller volume prolate particles, but larger volume oblate particles are taken up more efficiently than larger volume prolate particles (38). For endocytosis, simulated clathrin-mediated uptake is predicted to be at maximum efficiency for larger spherical particles, with optimal sizes close to the size limit of the relatively rigid clathrin lattice (more efficient uptake for 80 nm, than for 40 nm or 160 nm-sized particles (37)), and this also depends on ligand coverage and rigidity for simulated particles (36, 37). Finally, a theoretical study predicted that the curvature of the tip of the particle matters, as particles with high aspect ratios and round tips were predicted to enter *via* laying-down mode, while particles with smaller aspect ratios and flat tips might be ingested more efficiently in tip-first mode (46). This seems to contradict another computational modelling study, where it was predicted that, given that the particle rigidity remains the same, the uptake rate and membrane bending are similar for cubes and spheres, discs and oblate particles, and rods and prolate particles (**Figure 2B**) (39). Overall, a single general rule seems to apply to the uptake of such differently shaped particles: particles with larger volumes might be taken up more efficiently than particles with smaller volumes but the same AR (**Figure 3**), because of lower membrane curvature and a higher number of receptor-ligand interactions (38).

Concerning the mechanical properties of particles, computer simulations predict that the uptake of elastic (soft) particles is less efficient than of more rigid particles, because particle deformations lead to higher energy barriers for membrane wrapping, and uptake of softer particles might need more receptors for overcoming this energy barrier (**Figure 3**) (39). Uptake of oblate particles is predicted to be least sensitive to this elasticity if the particles are positioned tip-first because they have a larger contact area with the membrane promoting the recruitment of receptors facilitating uptake. Similarly, when a prolate particle is in laying-down position at the cell membrane, its uptake could be more efficient due to a larger contact surface area with the membrane (39). This may well have consequences for phagocytosis as well, because for example cancer cells can be less rigid and softer than normal cells (47), and perhaps their rigidity is a determining factor for being phagocytosed by cells of the immune system.

Finally, asymmetry of the membrane (i.e., different composition of the membrane leaflets) is predicted to strongly affect phagocytosis, as this causes spontaneous membrane curvature which can aid or hinder membrane wrapping and can form a membrane area reservoir for providing additional membrane for engulfment of particles (48).

These theoretical predictions have several caveats. First, in real biological systems, the interaction between particles and membrane can be heavily influenced by a “corona” of proteins on the particle surface, leading to non-symmetrical engagement of uptake receptors (49). In addition, cellular uptake can be influenced by sugars on the particle surface (50), a so-called “glycocalyx” consisting of glycoproteins, glycolipids, and proteoglycans on the surface of target cells (51) and pathogens (52). Second, the plasma membrane of immune phagocytes is a highly crowded environment with different receptors and many other membrane proteins clustered and segregated in functional domains, and modelling showed that the membrane distribution of receptors can affect phagocytosis (35, 36, 39, 41). Third, most modelling approaches have as main assumption that the energy for membrane deformation is the bottleneck for uptake, and do not incorporate the role of the cytoskeleton nor do they include regulatory feedback. While the wrapping of membrane around a particle has been predicted to be strongly influenced by competing membrane-cytoskeleton interactions (53, 54), simulations do not account for the active remodelling of the cytoskeleton that can actively promote, and might even be the bottleneck for, particle invagination (see below). Fourth, biological membranes contain large numbers of different components that do not interact with the particle, such as membrane proteins and (glyco)lipids, which might affect the local membrane curvature and are predicted by statistical mechanical modelling and molecular dynamics simulations to dramatically influence cargo uptake even if the total spontaneous curvature of a membrane remains unchanged (55). Fifth, quantitative comparisons of simulation and experimental timescales associated with the phagocytic process are problematic and can only be done qualitatively. Because of these caveats, experimental findings sometimes contradict theoretical predictions as will be discussed below.

## Model Particle Size and Shape: Experimental Evidence

Experimental studies with synthetic model particles also show that shape, size and rigidity are important determinants for phagocytosis, but, likely due to the high complexity in cells, experimental findings are not always in agreement with the predictions from theoretical modeling. Moreover, as will be detailed below, experimental findings between studies do not always agree with each other, likely due to differences in the cell type, modes of uptake and properties of the phagocytic cargoes. Nevertheless, from a comparison of these different studies, some general conclusions can be drawn on how particle size and shape affect the endocytosis and phagocytosis of model particles.

Model particles can be ingested by the same PRRs and uptake mechanisms as microbes. In the circulation, soluble antibodies and/or proteins from the complement system can be deposited on the surface of pathogens, called opsonization, and this triggers phagocytosis by Fc-receptors and the complement receptor 3 (CR3/integrin α_M_β_2_/Mac-1), respectively. Similarly, opsonization can mediate the uptake of model particles. For example, antibodies can also bind to the polyanionic surface of latex particles, which enhances their uptake by Fc-receptors, but the binding of antibodies (mainly IgG1 and IgM) may also trigger the classical complement pathway resulting in deposition of protein C3 cleavage products recognized by the complement receptors of host cells (56–58). Targets can also be recognized by a class of PRRs called scavenger receptors (SR), for example SCARB1 and MARCO, that recognize oxidized low-density lipoprotein (LDL) on the surface of apoptotic cells but also bind to polyanionic structures such as latex particles (56). Finally, integrins can mediate phagocytosis of particles (56, 59). Thus, naked polystyrene particles can be phagocytosed *via* four ways: i) direct binding to scavenger receptors, ii) opsonization by host-deposited IgG1 or IgM leading to iC3b binding followed by binding to complement receptor 3 (57), iii) opsonization by antibodies followed by recognition by Fc-receptors (FcR) (17), and iv) direct binding to integrins (α_V_β_3_, α_5_β_1_, and α_V_β_5_) (59). Particles composed of other materials are also ingested by immune phagocytes. For example, silica particles are recognized by scavenger receptors SCARA1, SCARB1, and CD36, while monosodium urate (MSU) crystals can directly bind to cholesterol of cell membranes facilitating internalization. MSU and cholesterol crystals can also activate complement pathways through crystal opsonization by complement factor iC3b (60). The uptake routes of particles are not always understood, and for instance the mechanism of uptake of hydroxyapatite crystals by macrophages is still unclear (60).

Concerning particle size and phagocytosis, the available evidence seems to suggest there is an optimal size for spherical particles of about ~3 µm in diameter for most efficient phagocytosis. Both *in vivo* and *in vitro* studies showed that rat alveolar macrophages can efficiently clear particles in the range of 1–5 μm (**Figure 3**) (61–63), with an optimal particle size of ~2–3 μm (63). This is in line with another study addressing uptake of smaller (<1 µm) particles of different shapes made out of a polyethylene glycol diacrylate (PEGDA)-based hydrogel by endothelial cells, where it was found that the larger and more oblate particles were ingested more efficiently than smaller and prolate particles by endothelial cells (26).

There is an upper limit in the size of particles that cells can ingest, as particles exceeding the size of macrophages can halt phagocytosis (64), and larger sized particles result in frustrated phagocytosis (22). For needle-shaped particles, this limit is typically around 15 µM (22, 65), but this depends on the cell type: Although long (>20 µm) polystyrene worm-shaped particles with aspect ratios of about 22.5 could not be completely internalized by rat alveolar macrophages (27), 20 µm long calcium carbonate (CaCO_3_) needles (aspect ratio >20) could be internalized by THP-1 macrophages and primary murine peritoneal macrophages (32). For spherical particles, a study comparing differently sized of poly(lactic-co-glycolic acid) (PLGA) particles orally administrated to mice showed that 10 µm size was the largest size of particles that could be phagocytosed by gut phagocytes (66), although this finding might also be explained by a lower penetration of larger particles through the mucosal barrier. Likely, cells cannot phagocytose very large particles, because this requires more membrane and an increase in membrane surface area, which might be approximately equal to the surface area of the target, can thereby limit the size of particles that can be taken up (67). There is a limit up to which the plasma membrane can stretch without rupture (68, 69), about 3% for red blood cells (68), and in order to meet the need of substantial membrane for enveloping the target particles, there are two types of sources for membrane: i) folds in the plasma membrane, and ii) intracellular vesicles and granules (70–74). It is quite likely that phagocytosis of large particles requires a trigger to regulate the mobilization of these membrane reservoirs, and the plasma membrane itself might provide a mechano-chemical tuning mechanism by generating membrane tension during the uptake (75). Indeed, membrane tension could be released in a sequential manner where first the plasma membrane surface area increases by ~20 to 40% due to smoothening of the folded membrane and subsequently additional membrane was provided by exocytosis near the phagocytic cup (76). Such delivery of intracellular vesicles also plays a role during later stages of phagocytosis, an internal source of membrane derived from lysosomes was shown to be crucial to maintain the membrane integrity of phagosomes containing expanding hyphae of *Candida albicans* (74).

Although the surface density of ligands for phagocytic receptors improves uptake overall, this also seems to depend on the size of the particles. For phagocytosis, higher IgG opsonization only enhanced uptake of polystyrene spheres between 0.5 and 2 µm by RAW264.7 macrophage-like cells, but not for spheres > 2 µm (18). When uptake of differently sized liposomes by alveolar rat macrophages and RAW264.7 cells was compared, the largest tested liposomes of 650 nm – 2 um were ingested more efficiently than smaller liposomes (77, 78). For clathrin-mediated endocytosis, the uptake of particles seems restricted by the size (~100 nm) of the clathrin lattice, and larger particles are ingested less efficiently (**Figure 3**). Seventy nanometer sized flat hexagonally shaped or spherical particles were taken up with similar efficiency by mouse alveolar macrophages, but already a slight increase in size to 120 nm resulted in a reduced uptake of hexagonally shaped particles (79). Similarly, 150 nm silica spheres were ingested more efficiently by RAW264.7 cells compared to 250, 500 and 850 nm spheres (80)and round silica nanoparticles of 70 nm were ingested more efficiently than 300 nm and 1 µm sized particles by the murine XS52 epidermal Langerhans cell line (81). Thus, in general, phagocytosis might be most efficient for a particle size of about 3 µm, while clathrin-mediated endocytosis is optimal for particles around 100 nm.

Theoretical predictions that spheres are more efficiently ingested than more extremely shaped particles (35, 38, 39) are supported by experimental evidence (**Figure 3**). For both endocytosis and phagocytosis, a wide range of particles was tested to prove that shape rather than size has more effect on cellular uptake, and uptake of spheres was more efficient than uptake of any other stretched shapes (25, 29). Similarly, in comparison with spherical shapes, elongated particles (derived from 150 nm or 2 µm PLGA spheres) were ingested less efficiently by J774.A1 macrophages (82). HeLa cells more efficiently endocytosed spherical over rod-shaped nanometer-sized gold particles (83). Micrometer-sized spheres made out of CdTe quantum dot-cysteine micro-composites were phagocytosed with higher efficiency than rectangular disks and especially needle-shaped particles by RAW264.7 macrophages (84). Another study reports that although prolate micrometer-sized polystyrene particles showed the best attachment to RAW264.7 cells, they were less efficiently phagocytosed compared to spheres (**Figure 3**) (30). The same study showed that not spheres, but oblate disc-shaped ellipsoid particles are internalized with the highest efficiency, but this difference disappears with increasing particle size (30). Compared to other shapes and sizes, polystyrene prolate ellipsoid particles with 2-3 µm in their longest dimension also attached more to mouse J774 and rat alveolar NR8383 macrophage-like cells, but offered no advantage in actual uptake (25, 31). A similar preference for attachment of prolate particles was shown for nm-sized silica cylinders over spheres, although extremely long particles (worms) attached less well to RAW264.7 cells (85). Finally, prolate PLGA particles were inefficiently phagocytosed by J774 mouse macrophages, but were phagocytosed as soon as the shape of the particles was changed to more spherical (3 µm diameter) by pressure and temperature (86).

Evidence suggests that phagocytosis of differently shaped particles requires different signaling cascades. Experiments with the amoeba *Dictyostelium* showed that the coordinated activity of the small-GTPases Rac and Ras at the phagocytic cup by the multidomain protein RGBARG (RCC1, RhoGEF, BAR, and RasGAP-containing protein) is key for uptake of particles and microbes of different shapes (87). The authors propose that RGBARG promotes the protrusion of the cytoskeleton at the periphery of the phagocytic cup by expanding Rac activation in this region, while it suppresses Ras at more central regions of the nascent phagocytic cup (87). Although *Dictyostelium* without RGBARG showed improved phagocytosis of larger model particles and yeast, the spatial regulation of Ras by RGBARG was found to be important for phagocytosis of elongated cargoes (87).

Concerning the mode of uptake in phagocytosis, experimental findings seem to contradict most theoretical predictions (**Figure 3**). Tip-first phagocytosis by NR8383 and J774 cells was shown to be more efficient for µm-sized flattened prolate particles (*i.e.*, particles with three different primary axes) (25) and for long worm-shaped particles with high aspect ratios (~22.5) (27), showing that uptake is fostered by membrane interactions with high positive curvature regions. The same was observed for uptake of 2 × 10 µm cylinders made out of multiple silica spheres glued together with agarose by murine alveolar macrophages (33). Interestingly, the phagocytic cup membrane was found to move along the length of the orthogonally positioned cylinders, showing that phagocytes were actively searching for high-curvature tips of the cylinders before uptake (33). Oblate µm-sized polystyrene particles were found to be phagocytosed inefficiently in tip-first mode by rat and mouse macrophage cell lines (25). A similar preference for uptake *via* high curvature (tip-first for prolate, or laying-down for oblate particles) over low curvature (laying down for prolate and tip-first for oblate particles) membrane contact was observed for rectangular disks composed of CdTe quantum dot–cystine microcomposites by RAW264.7 cells (84). Thus, all these findings show that phagocytosis is more efficient upon contact with higher membrane curved regions of particles and this seems to contradict most theoretical predictions (**Figure 3**) (35, 38).

Theoretical predictions that stiffer particles are generally better ingested by cells also do not always seem to hold true (39). A review of available experimental data reported on particle elasticity (39) showed that sometimes less rigid/softer particles are internalized with higher efficiency than more rigid particles. For example, phagocytosis of softer layer-by-layer (LBL) capsules by HeLa and SUM159 cells was found to be more efficient than more rigid counterparts (39). However, the opposite has also been reported and for instance antibody-opsonized deformable poly-AAm-co-AAc microparticles (DAAM-particles) with a relatively high rigidity of 7 kPa were phagocytosed by J774 murine macrophage-like cells with 6-fold higher efficiency than 1 kPa particles (19). This controversy is likely attributable to the different modes of uptake for different particles and cell types. For example, clathrin coats are relatively rigid and have a well-defined shape, and in some instances less-stiff particles might be easier to ingest because they can be deformed to better fit in clathrin-coated vesicles.

Particularly silica particles can be toxic to cells, although there does not seem to be consensus on the size dependency of this toxicity. In a study comparing uptake of 150–850 nm silica spheres by RAW264.7 cells, the larger particles caused higher toxicity as evident from higher membrane rupture, ROS and tumor necrosis factor (TNF)-α production (80). However, this contrasts findings in the mouse epidermal Langerhans cell line XS52, where 70 nm amorphous silica nanoparticles were more cytotoxic compared to 300 nm and 1 µm sized particles as assessed by acetate dehydrogenase (LDH) release (81). Moreover, crystalline 0.3 μm-sized silica particles caused more ROS and TNF-α production in RAW264.7 cells than larger 4.1 μm-sized particles (88). Also in non-phagocytic HeLa cells, spherical silica particles of 70 nm were proven to be more cytotoxic than 200 and 500 nm sized particles, and this was attributed to their higher tendency for lysosomal localization (89). The toxicity of silica is likely caused by inflammasome activation as will be discussed below.

## Pathogen Size and Shape: Experimental Evidence

Findings from model particles might not always be directly applicable to real pathogens, as pathogens can engage different phagocytic receptors resulting in different modes of uptake. Moreover, pathogens can sometimes remodel their shape and express virulence factors that can affect the phagocytic process (90–93). In this section, we provide an overview of how the shape and size of pathogens affects phagocytosis. The phagocytosis of differentially shaped and sized pathogens is best understood for fungi, which are therefore the main focus of this section.

**Figure 4:**
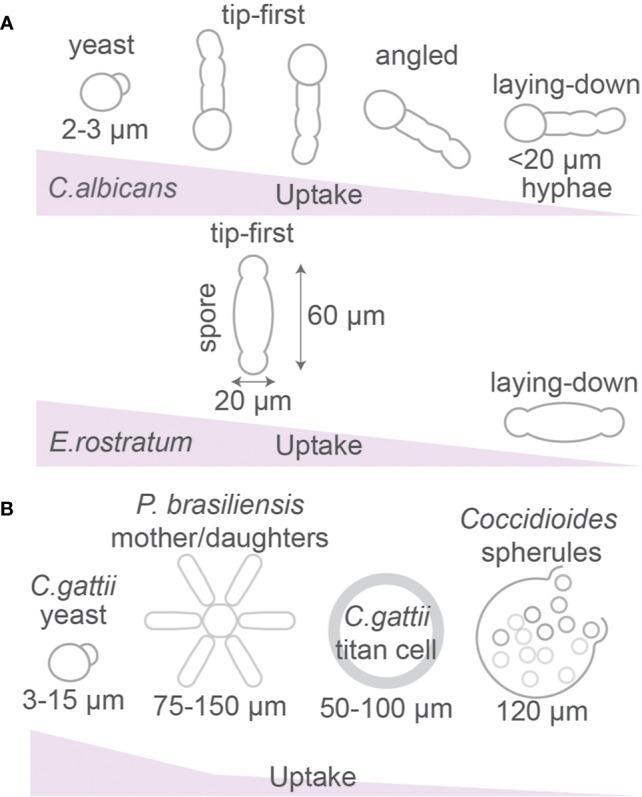
Fungal morphology affects phagocytosis. **(A)** Uptake efficiency of yeast and hyphal forms of *Candida albicans* and *Exserohilum rostratum* by macrophages. **(B)** *Paracoccidioides brasiliensis* can form star-like mother-daughter shapes. *Cryptococcus gattii* forms large titan cells and *Coccidioides* spp. forms spherules which are larger than the size of phagocytic cells. Details; see text.

Different strains of the pathogenic fungi *C. albicans* can have different morphologies, ranging from a spherical yeast to an elongated hyphal cell (**Figure 4A**) (7, 8, 13, 93–97). As predicted by modeling of spherical *versus* elongated particles (see above), live *C. albicans* with spherical morphology were engulfed more efficiently than hyphal *C. albicans*, and hyphae of above 20 µm (but not below 20 µm) significantly hindered uptake by J774.1 macrophages (7). However, phagocytosis of live yeast-locked mutant strains of *C. albicans* that cannot form hyphae (and therefore have more spherical shapes) was significantly slower than for strains that formed hyphae (7). The phagocytosis efficiency was also dependent on the orientation of the pathogen, because hyphal cells of *C. albicans* that were positioned toward a phagocyte in a tip-first orientation were taken up more rapidly than those engulfed at an angle or where cell-cell contact was in laying-down mode (**Figure 4A**) (7). Also for bacteria, phagocytosis of filamentous *Escherichia coli* bacteria by J774A.1 macrophages requires access to one of the terminal bacterial filament poles (tip-first), while a laying-down mode of uptake (with the longest axis of *E. coli* oriented parallel to the macrophage surface) was unsuccessful and no actin accumulation and phagocytic cup formation were observed (98). As discussed above, this contrasts previous theoretical predictions for the uptake of prolate ellipsoids, but is in agreement with experimental findings with model particles (**Figure 3**).

In contrast to elongated rods or filament shapes of bacteria that are taken up preferentially in a tip-first orientation, the phagocytosis of highly curved spiral-shaped bacterial species, for example *Campylobacter* and *Helicobacter*, might be stalled due to their shape (27, 99). Simulations have demonstrated inhibition in the uptake of such elongated particles in horizontal orientation due to the high energetic cost required for bending the plasma membrane around two different curvatures (99). Thus, the combination of a positive and negative curvature in spiral shaped particles may inhibit the membrane wrapping around the twisted surface and thereby prevent phagocytosis.

The hyphae of *C. albicans* are taken up more rapidly if phagocytosis of live fungi is initiated at the yeast-end than at the hyphae-end (7), perhaps because the round morphology of the yeast-end is more permissive for uptake. A similar observation was made for uptake of both live and heat-killed spindle-shaped spores of the fungus *Exserohilum rostratum* (~20 × 60 µm) (9). These are too large for uptake, but ~84% of macrophages attempted the uptake at the poles (reminiscent of tip-first uptake) and only ~16% positioned to the spore’s middle section (laying-down mode of uptake) (9). However, this differential macrophage positioning might not be attributable to the shape of the spores, but rather to differences in the cell wall composition at the poles and in the middle of the microbes (9).

Some *C. albicans* strains can alter their shape after phagocytosis, because *C. albicans* can avoid its destruction and evade immune response by forming germ tubes and hyphen within the sealed phagosome, resulting in perforation of the phagosomal membrane (**Figure 4A**) (95, 100). The formation of hyphae is promoted by the conditions within phagosomes, such as nitric oxide (101–103), H_2_O_2_ (104, 105), and alkaline pH (106). *Candida albicans* actively alkalinizes the phagosomal lumen by utilizing amino acids as a carbon source and excreting ammonia (94).

The fungus *Blastomyces dermatitidis* can adopt a “Trojan Horse” method where it first is phagocytosed efficiently in a small spore form (2–5 µm) by alveolar macrophages and later survives intracellularly by increasing its size 10-fold and disseminate from the host cell as a larger yeast form (10–30 µm) (107). This large yeast form can also be taken up by phagocytes, but less efficiently compared to the smaller spore form (107). Not only fungi, but also some bacteria can adopt a filamentous morphology in order to escape phagocytosis (108–110), but how this affects phagosomal maturation is incompletely understood.

As already mentioned above, spores (~20 × 60 µm) or hyphae of *E. rostratum* are too large to be phagocytosed by RAW264.7 macrophages and primary mouse bone marrow-derived macrophages (**Figure 4A**) (9). Similarly, the fungal pathogen *Cryptococcus gattii* is capable to form titan cryptococcal cells of 50–100 µm in diameter, which is larger than most immune phagocytes and prevents phagocytosis (13, 111), whereas smaller sized (5–10 µm) *Cryptococcus neoformans* particles are efficiently phagocytosed (112, 113). Other fungi such as *Coccidioides immitis* and *Coccidioides posadasii* also form giant spherules of about 120 µm to avoid uptake by host phagocytes. Once reaching the host organism, the pathogenic fungi *Paracoccidioides brasiliensis* and *Paracoccidioides lutzii* can impede phagocytosis by creating so called asteroid bodies which are round colonies with radial symmetry of mother cells (~30 µm^2^) surrounded by daughter cells reaching an area that ranges from 75 µm^2^ to over 150 µm^2^ diameter (**Figure 4B**) (114). Knock-down of the actin regulator Cdc42p in *P. brasiliensis* caused a size reduction of these asteroid bodies and promoted uptake by mouse bone marrow-derived macrophages and more efficient clearance from circulation after intravenous injection in mice, demonstrating that this pathogen increases its size to prevent host immune clearance (115).

**Figure 5:**
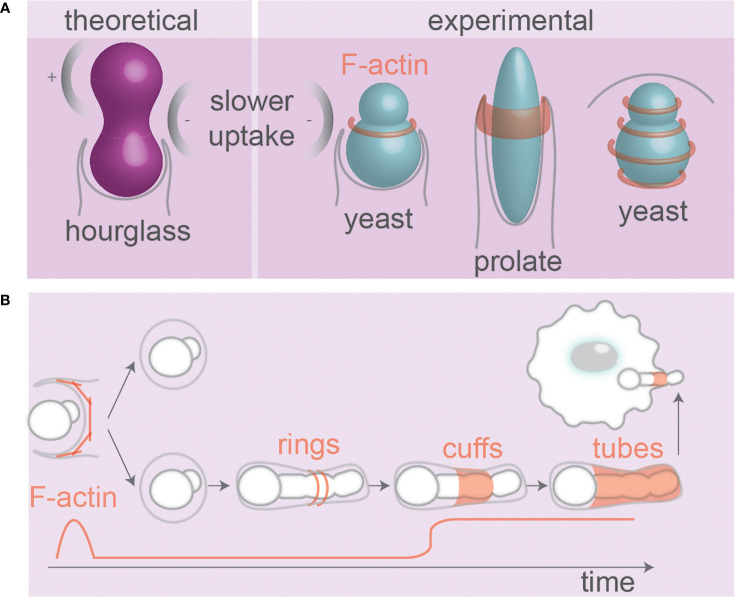
Pathogen and particle shape affect phagosomal F-actin. **(A)** Theoretical modeling of receptor mediated uptake of hour-glass particles and experimental evidence from budding yeast. The local negative curvature at the neck can slow uptake. Ring(s) of F-actin present during uptake of yeast (117, 118) and prolate particles (64). **(B)** F-actin at phagosomes containing fungi that alter morphology inside host cells. F-actin forms wave-like dynamic rings and cuffs around hyphae during internalization. At later stages, tubes of dynamic F-actin cover hyphae (93, 97, 119). Hyphal tips protruding membranes also recruit F-actin. Details; see text.

Evidence suggests that neutrophils can sense microbial size. Phagocytosis of living small yeast-locked *C. albicans* mutant strains triggers ROS production and elastase recruitment to phagolysosomes, whereas uptake of the larger hyphal forms of *C. albicans* or the large aggregates of *Mycobacterium bovis* lead to elastase translocation to the nucleus of neutrophils (6). The latter nuclear translocation of elastase causes proteolytic cleavage of histones and chromatin decondensation which leads to the release of NETs targeting pathogens (**Figure 1A**) (6). Finally, budding yeasts can stall phagocytosis when the phagocytic cup reaches the negative curvature at the neck separating a mother and daughter cell, as shown for phagocytosis of living yeast by the amoeba *Dictyostelium* (116) and for paraformaldehyde-fixed and heat-killed yeast by RAW264.7 macrophages (117). This is in line with findings from theoretical modeling that hourglass-shaped particles can stall phagocytosis (**Figure 5A**) (34).

## Phagocytosis and the Cytoskeleton

Phagocytosis is dependent on the actin cytoskeleton that aids in the protrusion of membrane for engulfment, forming a cup-shaped membrane fold around the particle (3, 17, 21) (**Figures 1** and **4B**). At the base of the phagocytic cup, F-actin is cleared thereby enabling the invagination of the membrane in a process driven by phosphoinositide remodeling (1, 2). At the rim of the phagocytic cup, F-actin rich pseudopodia continue to extent around the particle until they fully enwrap the particle and fuse, thereby sealing the particle into a phagosome (1, 2). The F-actin cytoskeleton exerts forces on the particles dragging them toward the center of the cell, and regulates membrane fusion events (118, 120–122). Moreover, the contractile activity of F-actin is necessary for phagocytic cup closure (17, 21). The role of F-actin in the phagocytic process has been extensively reviewed elsewhere (3, 17, 123–127), and we will limit our discussion to the role and organization of F-actin for uptake of differently shaped and sized phagocytic cargoes.

The organization of F-actin on the surface of phagosomes might well be dependent on the shape of the ingested particle. Super-resolution microscopy revealed that F-actin aligns in concentric rings or parallel fibers on the surface of phagosomes containing (spherical) zymosan yeast particles (**Figure 5A**) (118). So far only a limited number of studies addressed the question of how the F-actin skeleton is organized at the subdiffractional level on phagosomes containing particles/pathogens with irregular shapes (128), but since many proteins and lipids involved in cytoskeletal anchoring (integrins, phosphoinositides, ERM proteins) are sensitive to curvature, phagosomes carrying irregularly shaped particles can be expected to have distinct cytoskeletal-anchoring sites (129, 130). Moreover, because of the persistence length of F-actin filaments, it might be expected that F-actin filaments tethered to phagosomes containing elongated particles would align along the long axis of such particles. In line with this, *in vitro* experiments with polymerization of actin encapsulated in artificial liposomes converted the morphology of the liposomes from spherical to tubular (131). The notion that F-actin formation is dependent on particle shape is supported by the finding that F-actin only formed a ring at the leading edge of the phagocytic cup in alveolar macrophages phagocytosing spheres or flattened prolate particles (~14 × 4 × 1 µm) provided they were phagocytosed orthogonal to their major axis (tip-first) (**Figure 5A**). In contrast, even though F-actin was assembled if the initial contact with the particles occurred orthogonal to their minor axis (laying-down), it failed to form cup-like membrane extensions or a F-actin ring, which stalled uptake (64). Similarly, uptake of filamentous *E. coli* bacteria by J774A.1 macrophages was unsuccessful and no actin accumulation and phagocytic cup formation was observed when the bacteria were positioned with their longest axis parallel to the macrophage surface (laying-down) (98).

A study utilizing DAAM-particles with stiffnesses comparable to biological objects showed that, during phagocytic uptake by murine macrophage-like cells J774A.1 cells, pseudopods of phagocytes exert ring-like compression on the DAAM particles (19). Interestingly, those rings were not round and contained dents and pits indicating that the pressure was not uniform over the perimeter of the ring (19). The dents might be caused by localized pressure exerted by podosome-like spots of F-actin at phagocytic cups (132). During the uptake of the DAAM-particles, also pits caused by compressive forces were observed at the base of the phagocytic cup and these pits were even observed during phagocytic cup closure (19). It seems unlikely that these compressive forces are exerted by the F-actin cytoskeleton, as F-actin is cleared from the cup base at this stage of phagocytosis (133).

The F-actin cytoskeleton not only mediates phagocytosis, but also plays a role later during the maturation of the phagosome and fungal pathogens sometimes express virulence factors that interfere with this. For instance, the intracellular pathogen *Cryptococcus neoformans* can block acidification of its phagosomes in a pathogen-driven process and disrupt the integrity of the phagosomal membrane to gain access to the cytosol (134). This phagosomal permeabilization is followed by transient appearance of F-actin on the phagosomal surface during fusion of the phagosome with the plasma membrane, leading to non-lytic escape of *C. neoformans* from the cell (vomocytosis) (135). In addition to virulence factors, the size, shape, and/or surface properties of *C. neoformans* might also contribute to these actin flashes, as they were not only observed in phagosomes with live pathogen, but also with heat-inactivated pathogen and they were more persistent than for latex beads (135). A point of discussion is whether this F-actin has a protective function against phagosomal membrane rupture. Such F-actin flashes have also been observed on phagocytosed latex beads and heat-inactivated *C. neoformans*, but these flashes occurred with lower frequency and such particles were not capable of the non-lytic expulsion (135). Moreover, F-actin removal from the phagosome could be a necessary step before the phagosome can fuse with endosomes and lysosomes (117, 135), although this is controversial because the F-actin tethering protein ezrin was shown to stabilize phagosomal F-actin promoting the recruitment of the lysosomal membrane protein LAMP2 (136).

There are indications that the recruitment of F-actin during later stages of phagosomal maturation depends on the shape of the ingested particle. The neck of budding yeasts creates negative curvature between mother and daughter cells, which stalls F-actin in that region during phagocytic uptake by RAW264.7 macrophages, leading to persistent F-actin flashes in that region (**Figure 5B**) (117). F-actin was only present during the early stages of phagocytic uptake of *C. albicans* mutants that were unable to form hyphae, whereas it was also present after phagocytosis of wildtype hyphae-forming *C. albicans*. In fact, different topologies of F-actin have been observed on phagosomes containing the hyphal form of *C. albicans*: cuffs of F-actin at entry points (sleeve-like extended phagocytic cups, also observed in (97)), tubes of F-actin lining fully internalized hyphae (unlike the yeast form that does not have F-actin), and F-actin at hyphal tips (93) (**Figure 5B**). However, it is arguable how much of these differences in F-actin localization is driven by the geometry of the pathogen and how much it depends on cell wall composition; F-actin was less recruited to phagosomes containing *C. albicans* mutants with dysfunctional O-mannosylation (and disrupted cell walls), even though this mutant formed hyphae comparable to wildtype fungi (93).

Moreover, *C. albicans* might express virulence factors that alter F-actin formation at the phagosomes. Using the class I PI3K inhibitor LY294002, the activity of PI3K was shown to be unnecessary for later tube-like F-actin polymerization around *C. albicans* hyphae (97). Since class I PI3K is known to play an essential role in cortical F-actin dynamics (2, 4, 137), this suggests that *C. albicans* might trigger signaling cascades to evade host defense responses, either by creating a F-actin barrier independent from class I PI3K kinases that prevents fusion with lysosomes, or by utilizing F-actin for repairing the membrane rupture caused by fast growing hyphae. Interestingly, the role of PI3K in endocytosis (138) seems to depend on particle shape, as the broad PI3K inhibitor wortmannin could block uptake of nm-sized silica worms by RAW264.7 and alveolar macrophages, but affected uptake of spheres to a lesser extent (85).

Although much less studied, not only the F-actin cytoskeleton, but also microtubules might affect phagocytosis. First, microtubules control RAW264.7 macrophage spreading and migration, thereby linking this to phagocytic capacity (139). The involvement of microtubules depends on the phagocytic cargo, as phagocytosis of spherical 3 µm-sized non-opsonized and complement-opsonized silica particles by the mouse alveolar macrophage cell line MH-S, but not antibody-opsonized silica particles, depends on the microtubular cytoskeleton (140). Second, after phagocytosis, ingested particles are moved from the cellular periphery toward the microtubule organizing center (MTOC) located at the center of the cell (139, 141). This retrograde intracellular transport is likely mediated by motor proteins of the dynein family (142). Microtubules are thus responsible for the intracellular transport of phagosomes (143, 144) and this facilitates the fusion of phagosomes with endocytic organelles (145). In Fc-receptor mediated phagocytosis by RAW264.7 macrophages, the microtubules also re-orient the MTOC toward the phagosomal cargo, and this is necessary for Golgi positioning next to the phagosome for subsequent antigen presentation to T cells (141).

Actin and microtubules are also important for clathrin-mediated endocytosis and this depends on particle shape. For instance, it was shown that small molecule inhibitors of F-actin or inhibitors of microtubule assembly in macrophages caused a reduced uptake of nm-sized particles with more complex shapes, such as worms and cylinders, but not of spherical particles which were more sensitive to inhibitors of clathrin (85).

Since particle shape would be expected to determine the resistance and drag forces a phagosome encounters within a cell, it is logical to assume that particles will be reoriented within the cell to minimize the forces required for intracellular transport. In line with this, it has been reported that the initial contact is not the only parameter determining the direction of phagocytosis, and (at least spherical) particles can be rotated following uptake (146).

## Phagocytosis and Organellar Trafficking

Most studies on cellular uptake of differently shaped and sized particles only focus on the uptake mechanism, and not on the maturation of phagocytic/endocytic organelles within host cells. After full particle engulfment by the plasma membrane, both endosomes and phagosomes undergo a process called endo/phagosomal maturation. This maturation is driven by membrane fusion events, where compartments of early endosomal and lysosomal nature fuse with the endo/phagosomes, and by fission events characterized by vesicles budding-off from the endo/phagosome (1, 147–150). Cytosolic components, such as Rab proteins, their effectors, SNAREs, SNARE-interacting proteins, BAR-proteins, and phosphoinositide kinases, remodel the membrane of endo/phagosomes and thereby also drive maturation (1, 147, 148). After uptake of a microbial pathogen, the phagocyte attempts to kill the ingested pathogen by generating large amounts of reactive oxygen species (ROS) by the NADPH-oxidase NOX2 in the lumen of the phagosome (151). During endo/phagosomal maturation, the endo/phagosome becomes enriched in lipases, nucleases and proteases (126, 152). Among the proteases are cathepsins which play a role in antigen processing and MHC-class II chaperon chain pre-processing for antigen presentation to T cells (152). The resulting lysosome (endocytosis) or phagolysosome (phagocytosis) is a degradative compartment characterized by low pH, as a result of proton import by the vacuolar (v-)ATPase from the cytoplasm to the phagosomal lumen, and this enables the degradation of the ingested pathogen (1, 94, 108, 137).

The notion that membrane trafficking leads to asymmetry in phagosome maturation is well-established. This asymmetry can already occur early in the phagocytic process at the nascent phagocytic cup. For instance, it was observed that membrane fusion during phagocytosis of large spherical particles resulted in a displacement of the original membrane constituents at the base of the phagosomal cup, which thereby became more mature than peripheral regions and this heterogeneity was more apparent for larger particles (153). In line with this, a clear spatial gradient of phosphoinositide lipids and the proteins p85 and SHIP1 was observable early during phagocytosis of ellipsoid ~10 µm prolate polystyrene particles (154). In RAW264.7 macrophage-like cells, the uptake of PFA-inactivated filamentous *Legionella pneumophila*, *Salmonella typherium*, and *C. albicans* proceeded *via* long-lasting phagocytic cups and endosomes already fused with and released their contents along the surface of these nascent phagosomes (23, 155). However, despite acquiring late phagosomal markers (e.g., Rab7, LAMP1), the lumen of the nascent phagosomes did not acidify prior to sealing and low molecular compounds and lysosomal hydrolases could leak into the extracellular environment (23, 155). This shows that particle morphology affects phagocytic cup remodeling and closure, phagosomal acidification, and the integrity of the phagosomal membrane.

Also after sealing of the phagocytic cup, it can be expected that a maturation asymmetry is present on phagosomes carrying highly curved particles, since many phagosomal proteins (including BAR-domain proteins, vesicle coats) and lipids (phosphoinositides, phosphatidic acids) are responsive to membrane curvature (129, 130, 156, 157). Since these proteins and lipids regulate intracellular membrane fusion and fission, this raises the possibility that organellar trafficking events driving phagosomal maturation might predominantly occur at the highly curved membrane regions of phagosomes containing irregularly shaped particles. This might contribute to the pathogenicity of *C. albicans*, as it was found that phagosomes containing the hyphal form of the pathogen mature slower (93) and have prolonged presence of the small GTPase Rab14, which favors LAMP1 and cathepsin B recruitment compromising fungal survival and immune escape (119). In this study, the prolonged retention of Rab14 on phagosomes was found to be proportional to hyphal length, which supports the hypothesis that particle shape is a key parameter for phagosomal maturation.

## Inflammasome Activation

**Figure 6:**
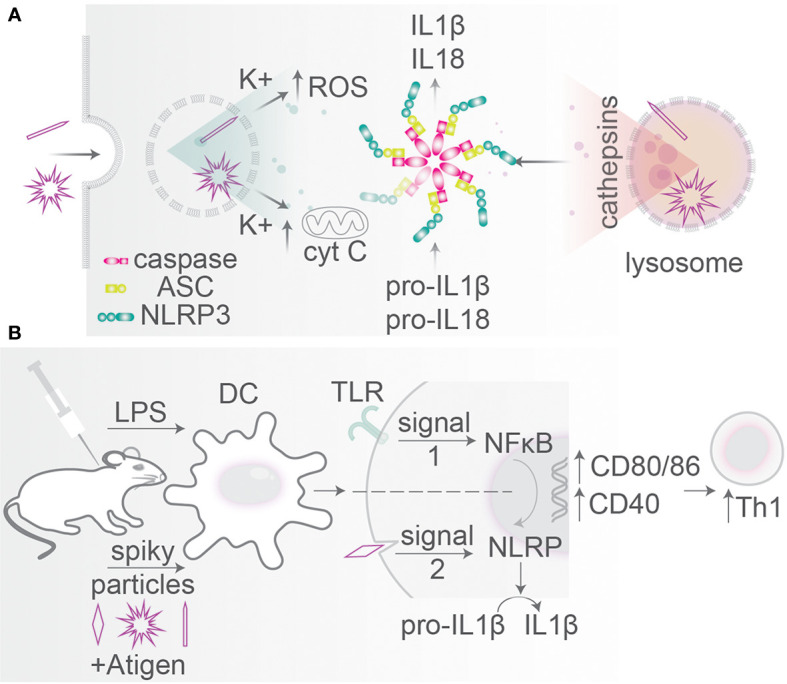
Particle size and morphology: inflammasome activation. **(A)** Particles with spiky morphologies can disrupt endo/phagosomal membranes leading to K^+^ and cathepsin leakage into the cytosol (so-called signal 2). Mitochondrial destabilization causes cytochrome C release leading to cytotoxicity. These stimuli activate inflammasome assembly from caspase-1, ASC, and NLRP3 which cleaves pro-IL-1β and pro-IL-18 into mature IL-1β and IL-18. Inflammasome activation can trigger cell death (pyroptosis). **(B)** Particle shape is an agonist in particle-based vaccination. Administration of TiO_2_ spiky particles initiating mechanical rapture of phagosomes (signal 2) in mice together with a bacterial TLR ligand (signal 1). TLR signaling activates NF-κB relocation to the nucleus leading to pro-IL-1β and pro-IL-18 transcription and translation and upregulation of activation markers CD40, CD80, and CD86. Signal 2 initiates inflammasome-mediated proteolytic conversion into mature IL-1β and IL-18. Mice receiving a combination of spiky particles and a TLR-ligand thereby induce a potent Th1 response.

Several MAMPs and DAMPs can be sensed by an important class of PRRs: nucleotide-binding domain and leucine-rich-repeat proteins (NLRPs) (158). Within the cytosol, NLRPs can assemble into a large macromolecular complex called the inflammasome which triggers caspase-1 activity. Caspase-1 causes production of the inflammatory cytokines IL-1β and IL-18, leading to inflammation and inflammatory cell death (pyroptosis) (**Figure 6A**). In various pathological conditions, crystals of alum (e.g., in vaccine adjuvants), urate (in the disease gout), cholesterol (atherosclerotic lesions), calcium pyrophosphate (pseudogout), asbestos (asbestosis, mesothelioma), and silica (silicosis) can be ingested by phagocytes and trigger inflammasome activation (158–160), and this is the main explanation why crystalline silica particles are cytotoxic (see above). Inflammasome activation can be caused by destabilization of the membranes of lysosomes leading to the release of cathepsin B into the cytosol and/or efflux of potassium ions through K^+^ channels (**Figures 6A, B**), which promote inflammasome activation and IL-1β and IL-18 production, but also can increase apoptosis due to cytochrome C release from mitochondria (158, 161–164).

**Figure 7:**
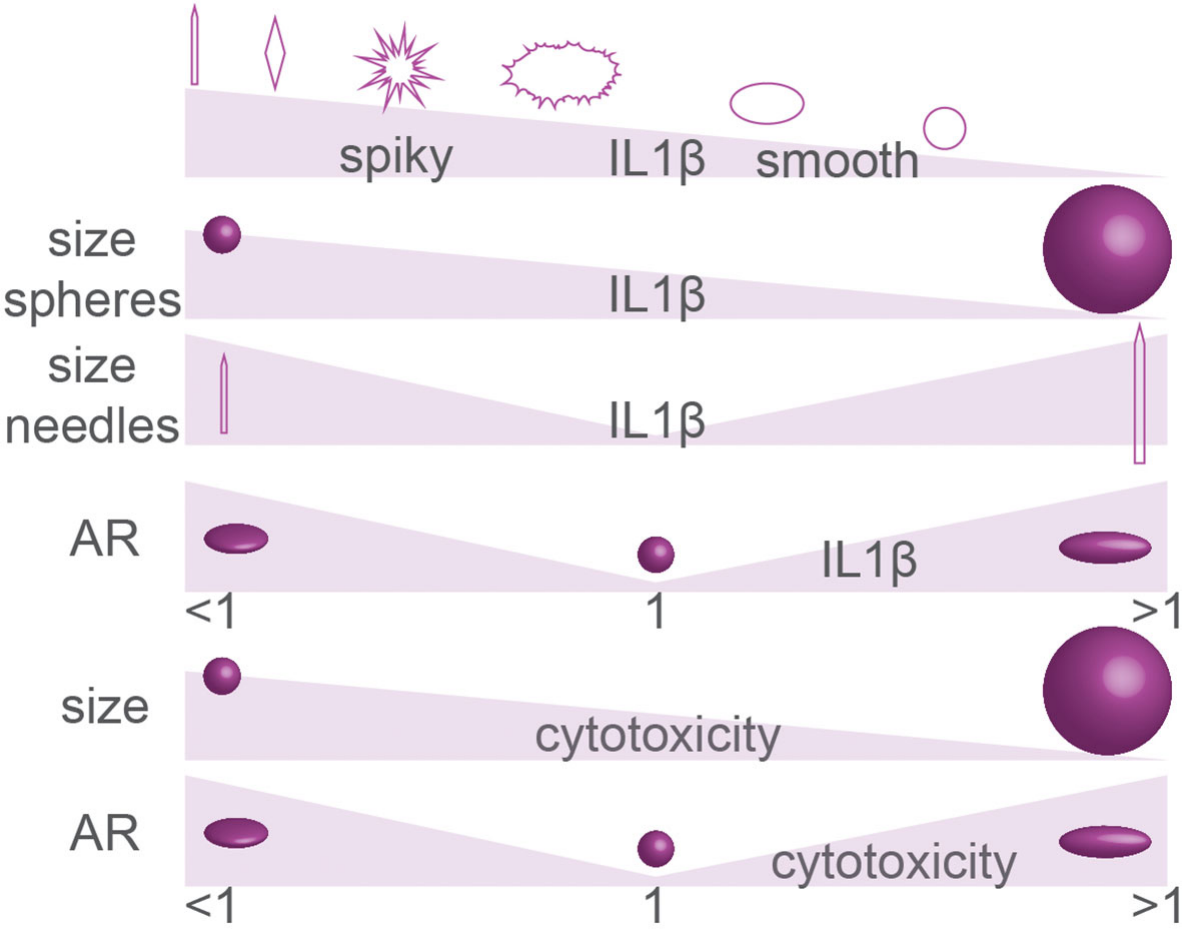
Summary of particle geometry in inflammasome activation (IL-1β production) and cytotoxicity. The effect of particle size on inflammasome activation and cytotoxicity is controversial. Details; see text.

The activation of the inflammasome by particles depends on their surface properties. Particles with reactive surface chemistry and/or particles with rough surface topologies (e.g., silica, carbon black, silver, polystyrene) can destabilize lysosomal and phagosomal membranes, thereby causing inflammasome activation, as has been extensively reviewed elsewhere (158, 165–168). Overall, for such particles with rough and/or reactive surfaces, smaller nanoparticles are more potent for inflammasome activation, because they have a larger surface area per mass, as also reviewed elsewhere (**Figure 7**) (166, 169). However, an inverse size-dependency has been reported for long particles with extreme AR. Such needle-like particles can also result in inflammasome activation, even if they are composed of biological inert material, and this is a hallmark of the fiber paradigm explaining the toxicity of long fiber like materials (asbestos, carbon nanoparticles) (22). For such particles with extreme NA, larger particles generally are more capable of inflammasome activation, as is well understood for needle-shaped particles of titanium rutile, poly(ethylene oxide), gold and carbon, and other materials (**Figure 7**) (161, 166). The activation of the inflammasome by needle-shaped particles depends on ROS production and cathepsin B (170), and although the precise mechanism and the contribution of membrane destabilization in this process are less clear (170, 171), it likely relates to frustrated phagocytosis and the leakage of ROS and lytic enzymes in the extracellular environment (the fiber paradigm) (22). Similar to needle-shaped particles, irregularly shaped particles with high curvatures can also induce the inflammasome and here larger particles are also more effective than smaller ones (161, 164, 166). For instance, 6 µm sized irregularly shaped CoCrMo particles were more potent for IL-1β production by human primary monocytes and human THP-1 monocytes than similarly sized smooth particles and 1 µm irregularly shaped particles (163).

The inflammasome can also be triggered following the uptake of certain pathogens. Here, the inflammasome can be activated following destabilization of endosomal or phagosomal membranes by virulence factors, but also certain topological cues such as the hyphae of fungi or some bacterial nanofeatures, such as flagella, pili, and fimbriae, might activate the inflammasome (74, 167, 168). For the pathogen *C. albicans*, this plays a role in its escape from phagocytes by lytic expulsion following pyroptosis (inflammasome-mediated cell death) (96). It is argued that the hyphae of *C. albicans* can trigger activation of the inflammasome which in turn triggers cytotoxicity of phagocytes (94), and that thereby the pathogen can escape from phagocytes. Whether filamentation is a sole trigger of such pyroptosis is debated, but it is agreed on that solely phagosomal rupture does not cause cell death, but rather a combination of NLRP3-activation, physical cell destruction, changes in pathogen morphology (96) and fungal cell wall remodeling (172) are required.

## Particle Size and Morphology in the Immune System

**Figure 8:**
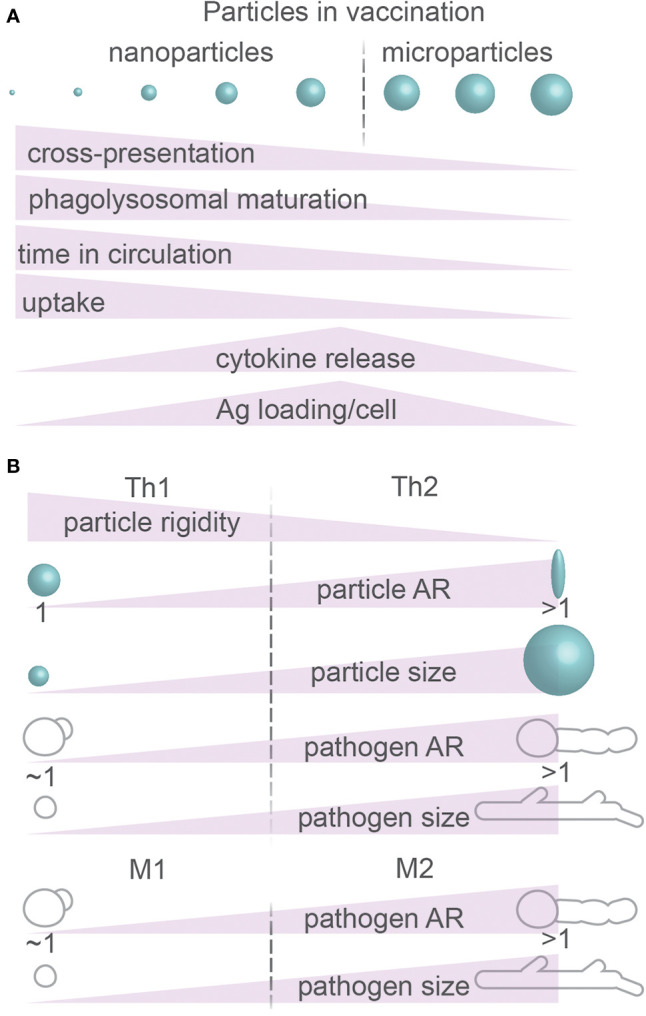
Particle size and morphology: immune responses. **(A)** Summary of immunomodulatory effects of physical properties of particles. Ag, antigen. **(B)** Summary of effects of physical properties of particles on CD4^+^ T cell differentiation (Th1 and Th2) and macrophage polarization (M1 and M2). Details; see text.

PRRs not only can mediate uptake but also signal intracellularly and IL-1β and IL-18 are critical for the immune system; it is therefore no surprise that particle size and shape can modulate immune activation as shown in both cultured immune cells and animal models (**Figure 8**). For example, compared to spherical particles composed of nucleic acids and cationic peptides, endocytosis by cultured mouse splenocytes of 10–100 nm sized fiber-shaped particles from the same material resulted in more expression of activation marker CD86 and the inflammatory cytokine IL-6, whereas expression of the co-stimulatory receptor CD40 and production of the inflammatory cytokine IFN-γ were reduced (173). In another study, the phagocytosis of 15–20 µm sized needle-shaped calcium carbonate particles by the THP-1 macrophage cell line resulted in more secretion of TNF-α and IL-8, thereby triggering a pro-inflammatory response (32). Finally, in mice injected with 7–8 μm-sized particles with complex geometries, neutrophil recruitment to the injection site was more rapid than with smooth particles, and phagocytosis, activation of the inflammasome and secretion of IL-1β were increased (174). The comparison of these *in vitro* and *in vivo* systems thus consistently shows that both endocytic and phagocytic cargoes with high NA induce more potent immune responses than more spherical particles.

The size of the particles is also an important determinant for immune activation. Hydroxyapatite crystals smaller than 1–2 µm caused more TNF-α release by cultured human primary macrophages compared to 6 and 14 µm sized particles (175), likely because of more efficient activation of the inflammasome as discussed above (166). However, the particle size also influences the processing of the antigens for activation of T cells. Antigenic proteins conjugated to nanometer-sized PLGA particles were more efficient in eliciting MHC-I cross-presentation in comparison to micrometer-sized particles in cultured DCs (**Figure 8A**) (reviewed in (176)). Here, the differently sized particles were found to be differently processed by the antigen presenting DCs: the proteins bound to nanometer-range particles were processed by the proteasome for cross-presentation in cultured DCs, indicating that they escaped from the lumen of the endosome into the cytosol (177). In contrast, proteins bound to micrometer-sized particles were processed by endolysosomal proteases (177), indicating that these proteins did not escape into the cytosol.

The size of the endocytic particles also influences the immune response by determining the speed of endosomal maturation. Endosomes containing smaller silver particles of 200 nm acquired lysosomal markers, such as LAMP1, sooner than endosomes carrying larger particles of 500 nm in cultured mouse bone marrow-derived macrophages (178). Although MHC-II was recruited to endosomes containing both types of particles, antigen presentation occurred more efficiently with the larger particles and this fostered more efficient CD4^+^ T cell activation (**Figure 8A**) (178). In this case, the efficient recruitment of lysosomes to endosomes containing smaller particles may result in a complete breakdown of the antigenic protein and thereby lower the efficiency of MHC-II presentation (179). Indeed, other studies found that not only the uptake but also the intracellular processing was determined by the particle size: 20–40 nm-sized particles composed of amphiphilic poly(gamma-glutamic acid) were not only more efficiently ingested by cultured murine bone marrow–derived DCs and RAW264.7 macrophage-like cells than larger 200–1,000 nm-sized particles (179, 180), but also underwent faster lysosomal degradation and were less likely to reside in earlier endosomal compartments (178, 179).

Because the shape and size of a particle can affect the immune system, it can affect the efficiency of particle-based vaccines (review (181)). First, the size of a particle is an important parameter for determining where a particle ends up in the body. Large particles (>1 µm) generally accumulate in small capillaries particularly in the lungs and can cause thrombosis (181). Partially due to uptake by macrophages and monocytes, which have an optimal particle size for endocytosis of about 100 nm as discussed above, particles with this size range are generally more readily cleared from circulation. For instance, one study reported that liposomal particles (bisphosphonates) carrying immune-activating molecules of ~80 nm size remained longer in circulation in animal models than 100 nm particles (**Figure 8A**) (78). However, the same study showed more potent immune responses for larger particles, as large liposomes (~200 nm) were more prone to cause cytokine production (IL-1β, TNF-α, IFN-γ, IL-6, IL-8, and IL-10) than small liposomes (~80 nm) both *in vitro* and *in vivo* (78). Finally, size is important for the efficacy of a vaccine, because large particles can simply contain more antigen than larger particles. PLGA particles of 40, 100, and 200 nm in size were administered subcutaneously in mice and, even although their uptake by lymphatic DCs showed a two-fold higher uptake for the smaller 40 nm particles, DCs carrying 200 nm particles showed a three-fold higher level of relative antigen content (180).

Based on different MAMP and DAMP signaling, DCs can discriminate between different pathogens and other immune challenges. This allows the DC to steer CD4^+^ T cells toward different subsets: Th1 or Th2 (182). Th1 cells secrete pro-inflammatory cytokines, such as IFN-γ and TNF-α, for activating MHC expression and antimicrobial responses in phagocytes. Th2 cells secrete more anti-inflammatory cytokines including L-4, IL-5, IL-10, and IL-13 for activating responses against extracellular pathogens *via* class switching and antibody production by B-cells. Moreover, IL-10 produced by Th2 cells inhibits the function and development of Th1 cells and macrophages (182). Because of these different cytokines, Th1 cells promote immune responses that favor microbial and viral elimination (10), while Th2 responses are more directed to fending off large parasites such as helminths and worms (182). In addition to recognizing different pathogens based on MAMP and DAMP signaling, and as discussed above, immune phagocytes can discriminate between different pathogen types based on shape and size and this plays a role in T cell differentiation (**Figure 8B**) (182). Although very small gold particles (2 nm; the size of a protein) do not efficiently activate DCs because they diffuse into cells and end-up in a non-membranous compartments, 12 nm-sized particles can enter the DCs *via* some form of receptor-mediated endocytosis and promote Th1 differentiation by the DCs (181). Moreover, OVA-conjugated polystyrene particles in size ~50 nm are more likely to initiate Th1 activation in mice than micrometer-sized particles (183, 184), whereas micrometer-sized particles are more prone to trigger Th2 responses (185, 186). In line with these findings, the smaller sized round conidia of *Aspergillus fumigatus* or yeast form of *C. albicans* skew immune responses by monocytes toward Th1, while the larger hyphae forms of these pathogens trigger more Th2 responses (**Figure 8B**) (10). However, this also depends on the shape and topology of the particles, particularly whether the inflammasome is activated as discussed above, because for instance nano-spikes on the surface of TiO_2_ particles resulted in Th1 responses in mice despite the large micrometer-range size of these particles (**Figure 8B**) (164).

## Future Prospects

An apparent conclusion in this review is that predictions from simulations and theoretical modeling do not always agree with experimental findings, particularly concerning the uptake mode of elongated oblate or prolate particles. As discussed above, most theoretical studies predict that such elongated particles are ingested by maximizing the contact area between the plasma membrane and the particle (laying-down mode for prolate particles; tip-first mode for oblate particles) (34–41). However, as also discussed above, this seems to directly oppose experimental findings with model particles and hyphen-forming fungal pathogens and filamentous bacteria (7, 9, 10, 23, 25–33, 93, 94, 96–98, 119, 181). A reason for this discrepancy might be that most simulations and theoretical modeling only/mainly consider the plasma membrane, and are therefore based on the assumption that the energy required for the remodeling of the membrane is rate-limiting for uptake. However, as explained in this review, different forms of endocytosis and phagocytosis are far more complex and this assumption might not hold true. In the future, more sophisticated models should incorporate this complexity and for instance account for regional signaling by uptake receptors, localized activation of Rho-GTPases and the polarization and active remodeling of the actin cytoskeleton.

**Figure 9:**
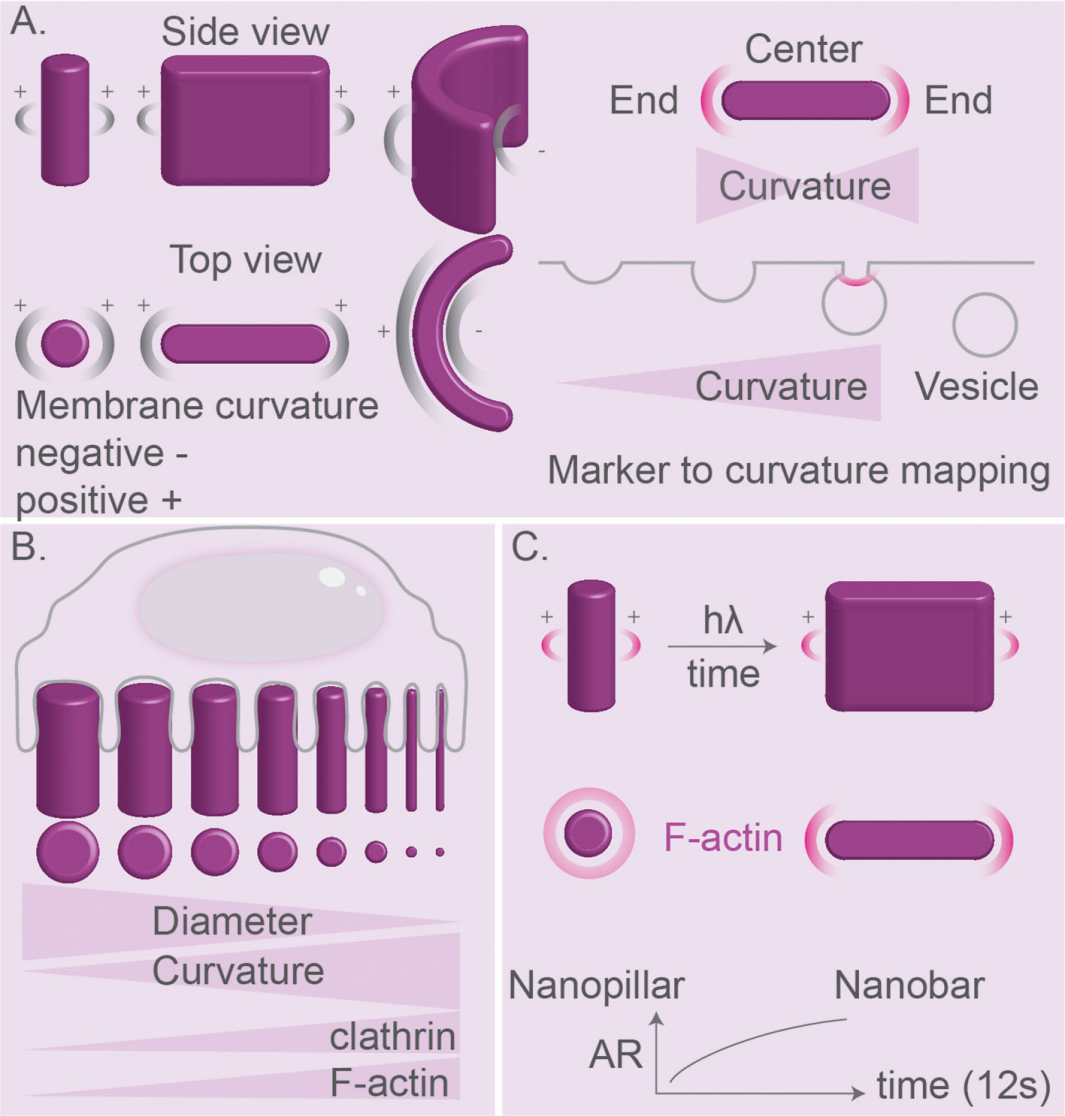
Nanostructure topologies for studying effects of curvature on membrane. **(A)** Nanostructure fabrication using electron-beam (E-beam) lithography. Pillars, bars, and C-like patterns can be designed for generation of a library of positive and negative curvatures and flat surfaces. Curvature libraries such as nano-bars can be used of mapping proteins sensing curvature in clathrin-mediated endocytosis. End-to-center ratio is higher for markers localizing mainly to the regions of high curvature. Curvature signature can be used for mapping of curvature-dependent protein recruitment in time. **(B)** Cells can be cultured on patterned substrates for nanoscale manipulation of membrane curvature. A smaller pillar diameter results in a higher curvature. Positive curvature with a radius of <200 nm is preferred by components of clathrin-mediated endocytosis and F-actin. **(C)** Azopolymers can change shape from vertical pillar to stretched nano-bar under the influence of light and can be used for dynamic manipulation of membrane curvature. Membrane stretching at nano-bar ends triggered F-actin nucleation.

As discussed in this review, many proteins and lipids involved in signaling and cytoskeleton-anchoring are sensitive to membrane curvature (129, 130, 156, 157, 187–189). Therefore, regions on particles with high positive or negative curvature might well result in phagosomal domains of protein/lipid interactions and signaling. These domains can be transient, given the highly dynamic nature of endocytosis and phagocytosis. In order to facilitate studying this, some approaches focus on creating stable membrane curvatures induced by surface topography that cells come in contact with (**Figure 9**) (188–192). Nano-topography-based methods such as electron-beam (E-beam) lithography can be used to create nanostructures as small as 50–100 nm, which enables high membrane curvatures (188–190). These approaches can be used for studying a wide range of questions related to curvature-induced distribution of clathrin-dependent endocytic proteins (187), polymerization of F-actin (188, 189) and nuclear membrane deformations affecting chromatin distribution and gene expression (190). Using this approach, it was found that nano-topographies of above 30 nm are sensed by macrophages and printed patterns above 71 nm in height induce higher endocytic activity (193). Moreover, microprinting allows creating adjacent structures with different sizes and topologies to compare these side-by-side using confocal or electron microscopy (**Figure 9B**) (187, 190). For example, one study using such a gradient of topologies showed that clathrin-related proteins prefer a positive curvature with a radius <200 nm (187). Moreover, an array of nanometer-sized bar-shaped structures with different diameters of the tips was used to show that only curvatures in the range of ~100–400 nm could trigger recruitment of the BAR protein FBP17 and the Arp2/3 complex required for F-actin assembly (188). An interesting modification of this method is the development of imprinted light-sensitive azobenzene-based polymer structures that can dynamically change their shape using light, thereby exerting forces and affecting the membrane curvature of cells interacting with these structures (**Figure 9C**) (189). Using this approach, it was shown that the light-induced elongation of vertical pillars, which generated a higher cell membrane curvature on the tips of the pillars, promoted the local accumulation of the actin nucleator Arp2/3 complex and F-acting assembly (189). This method may inspire more future approaches to dynamically manipulate the membrane curvature and analyze protein responses in real-time.

Other advances in the material sciences and microscopy will also lead to the development of better tools to study how particle size and shape affect different forms of endocytosis and phagocytosis. Modern synthetic methods allow to create functional colloidal building blocks and define general self-assembly principles (194). Particle’s shape, activity and patchiness can be engineered by combining classic sol-gel chemistry with a recently developed micro-emulsification methodology (195). These approaches will allow to design and fabricate biocompatible particles to serve as model phagocytic cargoes featuring tailored curvatures and symmetries. Designer cargos can be used for resolving particle reorientation within cells, heterogeneities of the phagosomal membrane, sites of membrane trafficking, and the arrangement of the cytoskeleton. Super-resolution stochastic optical reconstruction microscopy (STORM), stimulated emission depletion (STED) microscopy (128) and Förster resonance energy transfer-fluorescence lifetime imaging microscopy (FRET-FLIM) (196, 197) would be of benefit to determine membrane heterogeneities, the presence of maturation markers and cytoskeleton re-arrangements on the surface of irregularly shaped colloids engulfed by phagocytes. The combination of light sheet fluorescence microscopy with atomic force microscopy (AFM) enables to simultaneously visualize phagocytosis with force measurements (198) and hence will allow to answer the question of how particle size and shape affect the forces exerted by the cell. New image processing machine learning algorithms and digital holographic imaging techniques (128, 199) are gaining traction to visualize the positions and orientations of the colloids in 3D from 2D images. Phagosomes can be isolated from cells using density-gradient centrifugation or by using magnetic particles allowing for proteomic analysis. Such proteome analysis of different morphotypes of pathogenic fungi interacting with host immune cells may shed more light on the key curvature-sensing proteins that phagocytes rely on to interact with invaders (14).

In addition to these experiments with cells, experiments with artificial membranes can be of value and bridge the gap with modelling and theory. For instance, particles with a range of curvatures can be potentially encapsulated in artificial membranes for determining the organization of the membrane (*i.e.*, by encapsulating fluorescent lipid analogues, such as phospohoinositides, around the colloids for example by centrifugation of the colloids through a lipid/oil suspension on top of an aqueous buffer (200)). These artificial lipid membranes can be used for studying potential localized binding of recombinantly purified proteins that bind specific lipid species (e.g., pleckstrin-homology domains) or sense membrane curvature (e.g., BAR-domain proteins).

Current studies mostly employ either artificial colloids or killed microbial cells (by heat or chemicals) as models for phagocytosis, whereas the immune system encounters a wide range of pathogens in different shapes that actively move (e.g., by flagella) and can remodel their shape (13, 201). How pathogen motilities within phagosomes affect their degradation and whether the immune cell is somehow able to sense the movement of ingested pathogens is incompletely understood. As discussed above, various pathogens use shape to avoid clearance by the immune system, and the many pathogenic fungi can switch their morphology, for instance between small circular cells and extensive elongated hyphae (**Figure 5B**) (reviews (13, 201)). Moreover, bacteria of regular size (e.g., *Legionella pneumophila*, *Streptococcus pneumoniae*, uropathogenic *E. coli*) may undergo filamentation that slows down phagocytosis due to a higher AR (23, 98, 110). Size reduction is also used by some bacteria, as for example *Moraxella catarrhalis*, *Neisseria meningitidis*, *Salmonella typhimurium*, and *Streptococcus pneumoniae* convert from growing in longer chains to short chains, which results in a smaller surface area for complement deposition and reduced complement-dependent killing by phagocytes (110). These dynamic shape changes in the morphology of particles and the motion of particles in phagosomes can be studied using particles that can change their shape, for instance by light (189), and chemically powered micro- and nano-motors coupled to particles to induce self-propelled movements in fluids (202, 203) and by simulations (204). A particularly interesting class of model phagocytic cargoes are self-propelled colloids—synthetic particles equipped with a chemically powered nano-motor. The motor, typically platinum, catalyzes the decomposition of a suitable fuel generating a localized chemical gradient that propels the particle by either phoretic or bubble propulsion mechanisms (194, 205–211). Hematite or titania-based motors work in a similar manner, however, their activity can be controlled by light, thus allowing for a convenient on/off switch (212).

The H_2_O_2_ produced in phagosomes of neutrophils and dendritic cells by the NADPH oxidase NOX2 could be used as fuel to power active colloidal particles and generate forces in living cells (213, 214). More specifically, H_2_O_2_-fuelled particles might be engineered to utilize H_2_O_2_ produced in the phagosomal lumen by NOX2 for triggering particle motion, membrane rupture, and phagocytic escape (analogous to fungal pathogens). Moreover, membrane deformations by asymmetric forces exerted on the phagosome membrane by H_2_O_2_-fuelled active colloids might also result in polarized membrane trafficking. An alternative approach would be to create magnetic particles (*e.g.*, with Fe_2_O_3_ cores) to allow for external manipulation using magnetic fields (194, 205–211, 215).

Biocompatible particles with sizes in the nm range are of interest as carriers for drug-delivery or vaccination, therefore how their size, shape, rigidity, and surface composition affect cellular uptake is of particular interest (reviewed in (216)). Numerous studies attempt to use particles in the body to deliver drugs, vaccines, imaging agents or DNA or RNA for gene therapy (review (66, 164, 176, 181, 183, 184, 217)). Theoretical modeling and experiments addressing how particle shapes and sizes affect cellular uptake and (immune responses) might help in the design of such particle-based clinical agents (35–41).

## Concluding Remarks

-Theoretical simulations combined with experiments of pathogen shape, AR, and size may inspire particle design for vaccination for high antigen uptake or prolonged residence in circulation.
-Future theoretical studies should incorporate localized signaling of receptors and the cytoskeleton.
-The size and shape of pathogens affect the activation of antigen presenting cells and can skew T cell responses to Th1 and Th2 responses.
-Rough and spiky particles and particles with high AR can cause inflammasome activation and cell death.
-Pathogens change their shape to escape the immune system and this could be potentially used in vaccines.
-Molecular nanomotors can be used to study how pathogen mobility and shape-shifting affect phagocytosis and immune responses.
-Nano-topography-based methods of patterns printed on substrates can be used to gain a detailed understanding how curvature affects phagocytosis and different forms of endocytosis.

## Author Contributions

MB wrote the manuscript with support from MK, SS, ST, and GB. All authors contributed to the article and approved the submitted version.

## Funding

ST, SS and GB are funded by a Young Investigator Grant from the Human Frontier Science Program (HFSP; RGY0080/2018). GB has received funding from the European Research Council (ERC) under the European Union’s Horizon 2020 research and innovation program (grant agreement No. 862137) and the Netherlands Organization for Scientific Research (Vidi grant NWO-ALW VIDI 864.14.001). ST further acknowledges support from the Department of Atomic Energy, Government of India, under project no. 12-R&D-TFR-5.04-0800 and 12-D&D-TFR-5.10-1100, the Simons Foundation (Grant No. 287975) and the Max Planck Society through a Max-Planck Partner-Group.

## Conflict of Interest

The authors declare that the research was conducted in the absence of any commercial or financial relationships that could be construed as a potential conflict of interest.
