# Supplementary material for: Modulation of Immune Responses by Particle Size and Shape
Source: Front Immunol. 2021 Feb 12;11:607945. doi: 10.3389/fimmu.2020.607945 (PMC7927956; doi:10.3389/fimmu.2020.607945)
Supplement: Supplementary file 1 [file DataSheet_1.pdf]

Supplementary Table 1. Overview of a selection of theoretical studies addressing endocytosis.

Study	Type of modeling	Brief model summary	Parameters (selection)	Main conclusions (selection)
(Deng, Dutta et al. 2019)	Monte-Carlo simulations of ligand-receptor and clathrin-mediated endocytosis.	Nanoparticle uniformly covered with ligands. The nanoparticle is able to translate and rotate. The receptors can freely diffuse in the membrane.	80 nm spherical, oblate, prolate shapes.	Oblate particles: tip-first; near spherical slightly oblate or slightly prolate: tip-first and tilted; prolate particles: laying-down mode. Uptake of AR = 1 most efficient.
(Deng, Dutta et al. 2018)	Stochastic particle binding model and Monte Carlo simulations of ligand-receptor and clathrin-mediated endocytosis.	Nanoparticle uniformly covered with ligands. The nanoparticle is able to translate and rotate. The receptors can freely diffuse in the membrane.	40 to 160 nm spherical particles.	80 nm is optimal size for endocytosis.
(Deng, Dutta et al. 2019)	Stochastic particle binding model and Monte Carlo simulations of ligand-receptor and clathrin-mediated endocytosis	Nanoparticle uniformly covered with ligands. The nanoparticle is able to translate and rotate. The receptors can freely diffuse in the membrane.	80 nm spherical particles with range of ligand densities.	Optimal ligand density and binding affinity endocytosis.
(Chen, Xiao et al. 2016)	Coarse-grained molecular simulations.	Membrane bilayer with 18,000 lipids. Membrane rapping dynamics.	Particles spherical, prolate, oblate with different AR and volumes.	Small ellipsoids endocytosed in laying-down mode.
(Bahrami, Raatz et al. 2014)	Review of Monte Carlo and coarse-grained molecular dynamics simulations.	Elastic models of membranes to assess bending and adhesion energies during endocytosis	Reviewed different parameters for endocytosis.	Particle size affects adhesion energy. Membrane bending energy is minimal for AR=1. Oblate particles: laying down; prolate particles tip-first mode. Cooperative uptake of multiple particles in tube-like

				membrane invaginations.
(Shen and Ye 2019)	Coarse-grained molecular dynamics simulations.	Membrane of 27,000 lipids. Each lipid molecule is represented by a single spherical bead and nano-particles by several connected beads.	Elastic nanoparticles with different AR, elasticities, sizes (25 to 100 nm) and shapes (spherical and cubical, prolate and rod-like, oblate and disk-like).	Oblate shape is least sensitive to changes of particle elasticity during uptake. Oblate particles: tip-first; prolate particles: laying down.
(Richards and Endres 2016)	Numerical calculations of receptor mediated phagocytosis. Includes actin cytoskeleton.	Calculations of binding and membrane bending energies. Receptors fixed or mobile.	µm-sized spheres, ellipsoids, capped cylinders, and hourglass-shaped particles.	Membrane wrapping of highly curved regions less efficient. Some ellipsoids engulfing is faster than spheres. Phagocytosis of particles with high or too low AR stalled.
(Agudo-Canalejo 2020)	Calculations of free energies of endocytosis.	Differently oriented ellipsoidal particles relative to membrane. Membrane wrapping and particle adhesion energy.	Shapes: spherical, prolate, oblate expressed.	Spheres: optimal membrane engulfment, but membrane adhesion can be more efficient for ellipsoid particles.
(Bahrami 2013)	Monte Carlo simulation	Membrane is modelled as a triangulated vesicle with vertices.	Uptake of nm-sized particles in ~10-fold larger vesicles. Shapes: spherical, oblate, prolate.	Less efficient internalization for particles with high AR. Ellipsoidal particles reorient during uptake.
(Dasgupta, Auth et al. 2014)	Numerical calculations of endocytosis.	Triangulated membranes. Energy of membrane deformation, and particle adhesion.	Ellipsoid particles of 20-100 nm size with $1 < AR < 3$. Blunt tips and increasing edge curvature.	Increased AR is unfavourable for complete wrapping. High AR and round tips: laying-down mode; small AR and

				flat tips: tip-first mode.
(Khosravanizadeh, Sens et al. 2019)	Coarse-grained molecular dynamics model. Planar substrate mimicking the cytoskeleton.	Energy of membrane deformations and adhesion to particle and cytoskeleton.	Infinite long cylindrical nanoparticle (nanowire).	Initial membrane wrapping is independent of membrane tension, but complete wrapping is strongly dependent on membrane tension.
(Hashemi, Sens et al. 2014)	Calculations of free energies of endocytosis. Includes cytoskeleton.	Engulfment of a cylindrical object by a bilayer adhered to a rigid plane (cytoskeleton).	Cylindrical particles (100 nm - 1 µm radius).	Less efficient engulfment if interactions with cytoskeleton too strong.
(Curk, Wirnsberger et al. 2018)	Statistical mechanical modelling and coarse-grained molecular dynamics simulations of receptor-mediated endocytosis.	Calculations of binding and membrane bending energies and simulations of receptor with lipids (25 x 25 nm area). Intrinsic curvature of membrane system.	Differently sized spherical particles.	Endocytosis more efficient with negatively curved inclusions; lower with positively curved inclusions.

*AR: aspect ratio

References

Agudo-Canalejo, J. (2020). "Engulfment of ellipsoidal nanoparticles by membranes: full description of orientational changes." J Phys Condens Matter **32**(29): 294001.
Bahrami, A. H. (2013). "Orientational changes and impaired internalization of ellipsoidal nanoparticles by vesicle membranes." Soft Matter **9**(36): 8642-8646.
Bahrami, A. H., M. Raatz, J. Agudo-Canalejo, R. Michel, E. M. Curtis, C. K. Hall, M. Gradzielski, R. Lipowsky and T. R. Weikl (2014). "Wrapping of nanoparticles by membranes." Adv Colloid Interface Sci **208**: 214-224.
Chen, L., S. Xiao, H. Zhu, L. Wang and H. Liang (2016). "Shape-dependent internalization kinetics of nanoparticles by membranes." Soft Matter **12**(9): 2632-2641.
Curk, T., P. Wirnsberger, J. Dobnikar, D. Frenkel and A. Šarić (2018). "Controlling Cargo Trafficking in Multicomponent Membranes." Nano Letters **18**(9): 5350-5356.
Dasgupta, S., T. Auth and G. Gompper (2014). "Shape and Orientation Matter for the Cellular Uptake of Nonspherical Particles." Nano Letters **14**(2): 687-693.

Deng, H., P. Dutta and J. Liu (2018). "Stochastic simulations of nanoparticle internalization through transferrin receptor dependent clathrin-mediated endocytosis." Biochim Biophys Acta Gen Subj **1862**(9): 2104-2111.
Deng, H., P. Dutta and J. Liu (2019). "Entry modes of ellipsoidal nanoparticles on a membrane during clathrin-mediated endocytosis." Soft Matter **15**(25): 5128-5137.
Deng, H., P. Dutta and J. Liu (2019). "Stochastic modeling of nanoparticle internalization and expulsion through receptor-mediated transcytosis." **11**(23): 11227-11235.
Hashemi, S. M., P. Sens and F. Mohammad-Rafiee (2014). "Regulation of the membrane wrapping transition of a cylindrical target by cytoskeleton adhesion." Journal of The Royal Society Interface **11**(100): 20140769.
Khosravanizadeh, A., P. Sens and F. Mohammad-Rafiee (2019). "Wrapping of a nanowire by a supported lipid membrane." Soft Matter **15**(37): 7490-7500.
Richards, D. M. and R. G. Endres (2016). "Target shape dependence in a simple model of receptor-mediated endocytosis and phagocytosis." Proc Natl Acad Sci U S A **113**(22): 6113-6118.
Shen, Z. and H. Ye (2019). "Membrane Wrapping Efficiency of Elastic Nanoparticles during Endocytosis: Size and Shape Matter." ACS Nano **13**(1): 215-228.
